# Transcriptome Analysis of the Innate Immunity-Related Complement System in Spleen Tissue of *Ctenopharyngodon idella* Infected with *Aeromonas hydrophila*

**DOI:** 10.1371/journal.pone.0157413

**Published:** 2016-07-06

**Authors:** Yunfei Dang, Xiaoyan Xu, Yubang Shen, Moyan Hu, Meng Zhang, Lisen Li, Liqun Lv, Jiale Li

**Affiliations:** 1 Key Laboratory of Exploration and Utilization of Aquatic Genetic Resources, Shanghai Ocean University, Ministry of Education, Shanghai 201306, PR China; 2 National Pathogen Collection Center for Aquatic Animals, College of Fisheries and Life Science, Shanghai Ocean University, 999 Huchenghuan Road, Shanghai 201306, PR China; Chinese Academy of Fishery Sciences, CHINA

## Abstract

The grass carp (*Ctenopharyngodon idella*) is an important commercial farmed herbivorous fish species in China, but is susceptible to *Aeromonas hydrophila* infections. In the present study, we performed *de novo* RNA-Seq sequencing of spleen tissue from specimens of a disease-resistant family, which were given intra-peritoneal injections containing PBS with or without a dose of *A*. *hydrophila*. The fish were sampled from the control group at 0 h, and from the experimental group at 4, 8, 12, 24, 48 and 72 h. 122.18 million clean reads were obtained from the normalized cDNA libraries; these were assembled into 425,260 contigs and then 191,795 transcripts. Of those, 52,668 transcripts were annotated with the NCBI Nr database, and 41,347 of the annotated transcripts were assigned into 90 functional groups. 20,569 unigenes were classified into six main categories, including 38 secondary KEGG pathways. 2,992 unigenes were used in the analysis of differentially expressed genes (DEGs). 89 of the putative DEGs were related to the immune system and 41 of them were involved in the complement and coagulation cascades pathway. This study provides insights into the complement and complement-related pathways involved in innate immunity, through expression profile analysis of the genomic resources in *C*. *idella*. We conclude that complement and complement-related genes play important roles during defense against *A*. *hydrophila* infection. The immune response is activated at 4 h after the bacterial injections, indicating that the complement pathways are activated at the early stage of bacterial infection. The study has improved our understanding of the immune response mechanisms in *C*. *idella* to bacterial pathogens.

## Introduction

The grass carp (*Ctenopharyngodon idella*), with a long breeding history, is one of the most economical herbivorous freshwater fish species and, is widely distributed in Asia [1]. Due to its great growth performance, rich nutrient content, low cost of breeding and delicious meat, grass carp has become a dominant commercially farmed species, accounting for over 20% of freshwater aquaculture production in China [2]. Despite their excellent growth traits and large-scale breeding mode, grass carp are susceptible to various diseases. Outbreaks of disease associated with bacterial pathogens, such as *Aeromonas hydrophila*, have caused high mortality, resulting in a decline in production and tremendous economic losses; these factors severely restrict the development prospects of the grass carp farming industry [3–6].

To date, no excellent culture varieties of the grass carp have been obtained by traditional cultivation techniques. Because the filial generation has a long sexual maturity period, the hybrid breeding strategy is not viable. Moreover, because of a failure to understand the genetic background of grass carp, little molecular breeding technology has been applied [7]. Hence, research is required to establish a useful genetic breeding program; for disease control, further research into the grass carp’s immune system, to identify immune-related genes and pathways, would be beneficial [8].

*A*. *hydrophila* is a pathogenic organism that causes a broad spectrum of diseases in humans and animals [9]. While it was thought to be an opportunistic pathogen in humans, a growing number of intestinal and extra-intestinal cases of disease indicate that *A*. *hydrophila* is an emergent human pathogen, irrespective of the immunologic aspects of the host [10]. *A*. *hydrophila* is a Gram-negative motile bacillus that causes motile aeromonad septicemia [11]. Therefore, effective measures are required to protect against infections with this pathogenic micro-organism in aquatic animals. The identification of differentially expressed genes (DEGs) following infection with *A*. *hydrophila* is an important process towards understanding more about fish motile aeromonad septicemia.

Next-generation sequencing (NGS) technology for transcriptome processing based on high throughput RNA sequencing (RNA-Seq), including Illumina, can be used to generate a large amount of sequence data for organisms [12]. Using this sequencing method, the relative abundances of immune-related genes within several fish and mammalian species have been studied; species studied include the Tasmanian devil (*Sarcophilus harrisii*) [13], Chinese giant salamander (*Andrias davidianus*) [14], naked carp (*Gymnocypris przewalskii*) [15], and turbot (*Scophthalmus maximus*) [16]. Exploring the genome resources of an organism provides an opportunity to analyze the structural and functional genes related to the immunity responses in that organism [17–20]. However, expressed sequence tags (ESTs) have not yet been used to identify the gene expression profiles related to the bacterial immunity response in *C*. *idella*.

In present study, we used Illumina NextSeq^™^ 500 [21–26] sequencing to describe the transcriptomes of seven sets of spleen samples taken from fish exposed to either *A*. *hydrophila*, or not. The aims of the study were to (1) provide the most complete gene sequence resources data for the grass carp to date; and (2) identify the immune-related differential expression of genes in the complement and coagulation cascades and complement-related pathways. We believe that the knowledge of these genes and the transcriptome data produced will greatly facilitate breeding programs and genome-wide association studies for the grass carp.

## Materials and Methods

### Ethics statement

All experiments with fish in this study were conducted in accordance with the guidelines on the care and use of animals for scientific purposes, set up by the Institutional Animal Care and Use Committee (IACUS) of Shanghai Ocean University, Shanghai, China. The IACUS approved this study within the project “Breeding of Grass Carp” (approval number is SHOU-09-007).

### Experimental treatments and sample collection

According to the result of medial lethal concentration of the pre-experimental, Disease-resistant and common species of healthy grass carp (70 ± 2.3g, n = 160; about 12 months old), were obtained from the Wujiang National Farm of Chinese Four Family Carps, Jiangsu Province, China. Animals were cultured at 28 ± 0.5°C in a circulating water system sterilized with potassium permanganate, in which the water was aerated for two weeks before the experiment. The fish were fed three times per day (8:00 am, 12:00 pm and 5:00 pm) with 5% of their total body weight. The *C*. *idella* specimens were then randomly separated into four tanks (40 individuals in each tank). Fish in the first two tanks were given 100μL intra-peritoneal injections of *A*. *hydrophila* (AH10; Aquatic Pathogen Collection Center of Ministry of Agriculture, China) that was suspended in 1 × PBS at a dose of 2.7 × 10^7^ CFU/mL. Fish in the other two tanks were injected with the same volume of 1 × PBS, as a control. After injection, all the animals were raised under the same felicity conditions. Three fish were randomly selected from each control tank at 0 h and from the experimental tank at 4, 8, 12, 24, 48 and 72 h. The fish were euthanized with 100 mg/L of MS-222 (tricaine methanesulfonate; Sigma, St. Louis, MO, USA) and maintained on ice before tissue collection. The spleen samples were taken from the specimens and were immediately frozen in liquid nitrogen. The samples were then stored at −80°C until isolation of the total RNA could be carried out. The total RNA was used to sequence the transcriptome of the specimens.

### RNA isolation

Total RNA was extracted using TRIzol Reagent (Invitrogen, Carlsbad, CA, USA), following the manufacturer’s instructions. Then, DNA I (Thermo Scientific, USA) was used to remove the genomic DNA. All the described steps were carried out in a Vertical Superclean Bench. The quantity and quality of the RNA from each sample was determined using a Nanodrop 2000C spectrophotometer (Nanodrop Technologies, USA) and agarose gel electrophoresis, respectively. High quality RNA, with absorbance OD_260_/OD_280_ ratios of 1.8–2.0 and OD_260_/OD_230_ ratios ≥2.0 was obtained; it was clear and the brightness of the 28S:18S was close to 2.0. This RNA was used to construct the cDNA library.

In preparation for the construction of the cDNA library, the total RNA of each sample was standardized to 200 ng/μL. Then, the standardized volume of total RNA extracted from the spleen samples from the same time point (n = 6) were combined into one pool. This produced seven RNA pools (one for each time point). Each RNA pool was processed with Turbo DNA-free (Ambion, Austin, TX, USA) and was purified using the RNeasy Mini Kit (Qiagen, Valencia, CA, USA), according to the manufacturer’s instructions. The RNA quantity and quality were determined once again.

### mRNA purification, cDNA library construction and sequencing

The seven cDNA libraries of the spleen RNA samples were constructed using Illumina TruSeq^™^ RNA Sample Prep Kit (Illumina, San Diego, CA, USA), according to the manufacturer’s instructions. Poly(A) mRNA from each time point was isolated and purified from the total RNA with magnetic oligo-dT beads. The mRNA was first composited with divalent cations, and was then heated to obtain short fragments of about 200 bp. First-strand cDNA was synthesized from the short fragments using reverse transcriptase and random primers. Then, it was transformed into double-stranded cDNA using DNA polymerase I and RNase H.

The paired-end libraries were constructed with the double-stranded cDNA using a Genomic Sample Prep Kit (Illumina, San Diego, CA, USA). Each library was purified using the QIAquick PCR Extraction Kit (Qiagen, Valencia, CA, USA) and linked with sequencing adaptors after end repair of the double-stranded cDNA. Unsuitable fragments and the adaptors were removed using AMPure XP beads (Beckman Coulter, Shanghai, China). Then, each sequencing library was amplified by polymerase chain reaction (PCR). PicoGreen (Quantifluor^™^-ST fluorometer E6090, Promega, CA, USA), fluorospectrophotometry (Quant-iT PicoGreen dsDNA Assay Kit; Invitrogen, P7589), and an Agilent 2100 (Agilent 2100 Bioanalyzer, Agilent, 2100; Agilent High Sensitivity DNA Kit, Agilent, 5067–4626) were used to check the quantity, length and the distribution of the fragments in each cDNA library. The seven multiplexed cDNA libraries for each treatment were normalized to 10 nM. Then, the libraries were diluted to 4–5 pM, quantified, and sequenced using the Illumina NextSeq^™^ 500 platform (Shanghai Personal Biotechnology, Shanghai, China).

### Data filtering and de novo sequencing assembly

The mean length of the raw data across the seven cDNA libraries was about 150 bp. To obtain high-quality clean data, the adapter sequences were removed from the original reads, and a 5-bp window was used to check the raw data, from the 3’ to 5’, trimming the low-quality sequences with a Q score <20. Any sequences for which the final length was less than 50 bp were removed. In order to get accurate and comprehensive unigenes, the clean data of the seven transcriptomes for each treatment were assembled, because this process tends to eliminate genome-wide reference from the same species [27]. After filtering the raw data, the quality of the sequences was controlled and analyzed using FastQC (http://www.bioinformatics.babraham.ac.uk/projects/fastqc/).

Trinity software (http://trinityrnaseq.sf.net) was used to perform the *de novo* transcriptome assembly [28]. Using Inchworm, a K-mer library was built with the filtered reads to form contigs; a component was constructed with the contigs using Chrysalis and then a De Bruijn graph was generated. Butterfly was used to optimize the De Bruijn graph and establish transcripts through the paths [29]. All the transcripts were searched against in the NCBI non-redundant (NR) database (http://ftp.ncbi.nlm.nih.gov/blast/db/) using the BLASX algorithm (E-value≤1E-05). The unigenes were obtained after clustering the top-hit results. In order to ascertain the sequence directions of the unigenes that could not be aligned from the databases, ORF Finder [30]software was used to predict their open reading frames, with default settings, except that the parameter “-find,” was set as “1”.

### Gene functional annotation and classification

For a further functional understanding of the unigenes, Gene Ontology (GO) (http://www.geneontology.org/) annotations were determined using the Blast2 GO program [31–34]. In order to obtain the common denominators or functional categories of the unigenes, they were also annotated with the eggNOG database (http://www.ncbi.nlm.nih.gov/COG/, http://eggnog.embl.de/version_3.0/). The KEGG database (http://www.genome.jp/kegg/) was used to achieve pathway annotations and the KEGG mapper (http://www.genome.jp/kegg/tool/map_pathway2.html) was used to identify DEGs that the pathways showed.

### Comparative expression analysis

Reads per kilobase per million mapped reads (RPKM) are widely used as expression values for DEGs in RNA-Seq [35]. However, we used a more precise method to predict those expression levels in this study [36]. The DEGs were identified using DESeq (http://www.huber.embl.de/users/anders/DESeq), a fold change of <0.5 or >2.0 (that is, 2-fold up- or down-regulated), with a *p*-value < 0.05, was considered as significant differential expression [36–39]. The RPKM values of those DEGs were then considered as the expression levels, based on the number of reads aligned to each gene. Comparative expression of the genes was visually displayed using a Volcano Plot. Volcano plots are usually used to represent the microarray of mRNA expression levels.

TreeView (http://bonsai.hgc.jp/~mdehoon/software/cluster/software.htm/, http://jtreeview.sourceforge.net) [40], Cluster 3.0, MeV [41], and Java TreeView [42] software packages were used to perform cluster analysis of the gene expression patterns. We screened the DEGs of the spleen tissue and used Blast2 GO to perform GO enrichment analysis. The *p*-value denoted the magnitude of the difference in expression. The GO classification information of the functional unigenes from the spleen tissue was obtained through comparison with the entire genome database of other teleost fish. KO analysis was performed using the expression levels of the DEGs (up- or down-regulated). The KEGG pathways (http://www.genome.jp/kegg/tool/map_pathway2.html) were used to indicate the location of the DEGs in the different pathways. Pathway classification enrichment analysis of the DEGs showed the differences between the genes based on the *p*-values.

### Quantitative real-time PCR verification

To validate the transcriptional level of the key DEGs and the quality of the RNA-Seq, samples from separate specimens at each time-point of the experiment were collected. 30 unigenes were selected for quantitative real-time PCR (qRT-PCR). qRT-PCR was performed using a CFX96^™^ Real-time PCR Detection System (Bio-Rad, USA), with SYBR Green Master Mix (Takara, Shanghai, China) as a fluorescent dye, according to the manufacturer’s instructions. Primers were designed according to the RNA-Seq results, using Primer Premier 5 (Premier, Canada) (S1 Table) [43]. The arithmetic mean value of the 18S rRNA [44–46] and *β*-actin [47–49] genes of grass carp served as internal control value, to normalize the expression levels. After total RNA from each sample at each time point was extracted, reverse transcription was used to synthesize cDNA using the PrimeScript^™^ RT reagent Kit with gDNA Eraser (Takara, Shanghai, China), according to the manufacturer’s instructions. The reaction was performed in a total volume of 20 μL, including 10 μL of SYBR Green Master Mix (Takara, Shanghai, China), 1 μL of diluted cDNA mix, 0.6 mL of each primer (10 mM), and 7.8 μL of RNase-free water. The thermal profile for the SYBR Green RT-PCR was 95°C for 20 s, followed by 40 cycles of 95°C for 5 s, 55°C for 30 s, and 72°C for 30 s. First, a standard curve was used to assess the accuracy of primers, of which the amplification efficiency between 95% and 105% were chosen for candidate primer. Then, amplification and detection of only one PCR product was confirmed using a melting curve analysis of the amplification products at the end of each PCR. All experiments were performed in triplicate, providing technical repeats. Expression levels of the different genes were analyzed using the comparative CT method (2^−ΔΔCT^) [50].

## Results and Discussion

Identification the immune-related genes involved during the resistance of disease, such as those caused by *A*. *hydrophila*, provides a theoretical basis for disease prevention in teleost fish. Moreover, understanding the expression patterns of the genes involved in the response to motile aeromonad septicemia is the first step towards understanding the molecular mechanisms in grass carp. Qualitative and quantitative analysis of the immune regulation signal pathways, mediated by the candidate genes, also provide the possibility of enhancing non-specific immune-related enzyme activities in *C*. *idella*.

### *De novo* assemblies and data analysis

The raw reads of the seven transcriptomes have been deposited in the NCBI Sequence Read Archive (SRA) database (http://www.ncbi.nlm.nih.gov/Traces/sra/; accession number SRP060308). From the RNA sequencing, a total of 149.51 million 150 bp paired-end raw reads were generated, with an average of 21.36 million reads for each of the samples (S2 Table). After trimming, 122.18 million clean reads remained, with 78.11%–85.25% of the reads per sample being useful, equivalent to 32.85 GB clean data (S3 Table). The clean reads were assembled into 425,260 contigs, with a mean size of 310 bp and N50 length of 412 bp for 46.98% of them. They generated 191,795 transcripts, with an average length of 694 bp. 52,668 unigenes were generated, with an average length of 1,072 bp, ranging from 201 to 17,133 bp (Table 1). Approximately 91.25% of the contigs were <599 bp and 60.06% were 100–199 bp; 70.52% of the transcripts were 100–599 bp; and 98.69% of the unigenes were 200–4,999 bp (S1 Fig).

**Table 1:** Summaries of cDNA libraries sequencing of grass carp by Illumina NextSeq 500 platform.

	Contigs	Transcripts	Unigenes
Total length (bp)	131,702,960	133,136,713	56,482,473
Sequence No.	425,260	191,795	52,668
Max Length (bp)	18,447	17,133	17,133
Average Length (bp)	310	694	1,072
N50	412	1,142	1,787
>N50 Reads No.	61,288	28,702	9,318
GC%	46.98	47.88	49.72

**Fig 1:**
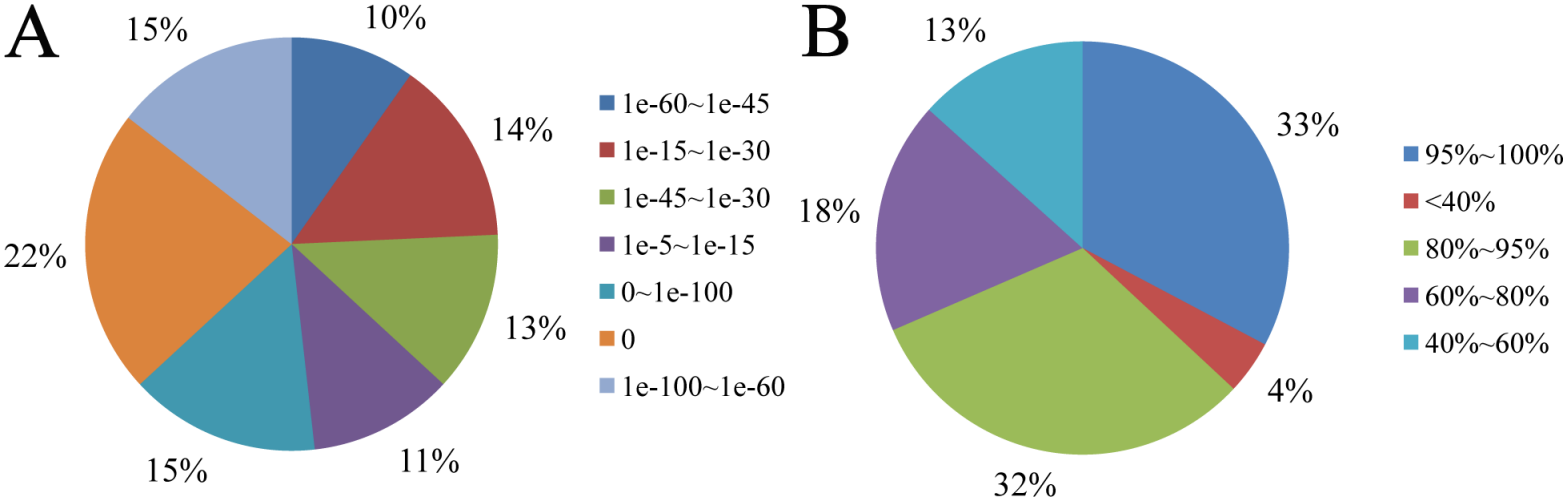
Unigene distribution in the *C*. *idella*. (A) E-value distribution of unigenes annotated into public databases with an E-value cut-off of 1E-50. (B) Identity distribution of unigenes annotated into public databases with an E-value cut-off of 1E-50.

The E-value results showed that 85% of the sequences had a strong homology with their expected distribution (<1.0 e-50) (Fig 1A); 18% of the sequences were between 60% and 80%, while 65% were over 80%, similar with their expected distribution patterns. Thus, 83% of the transcripts had a distribution higher than 60% of the E-value, which supported the credibility of the *de novo* assembly (Fig 1B).

**Fig 2:**
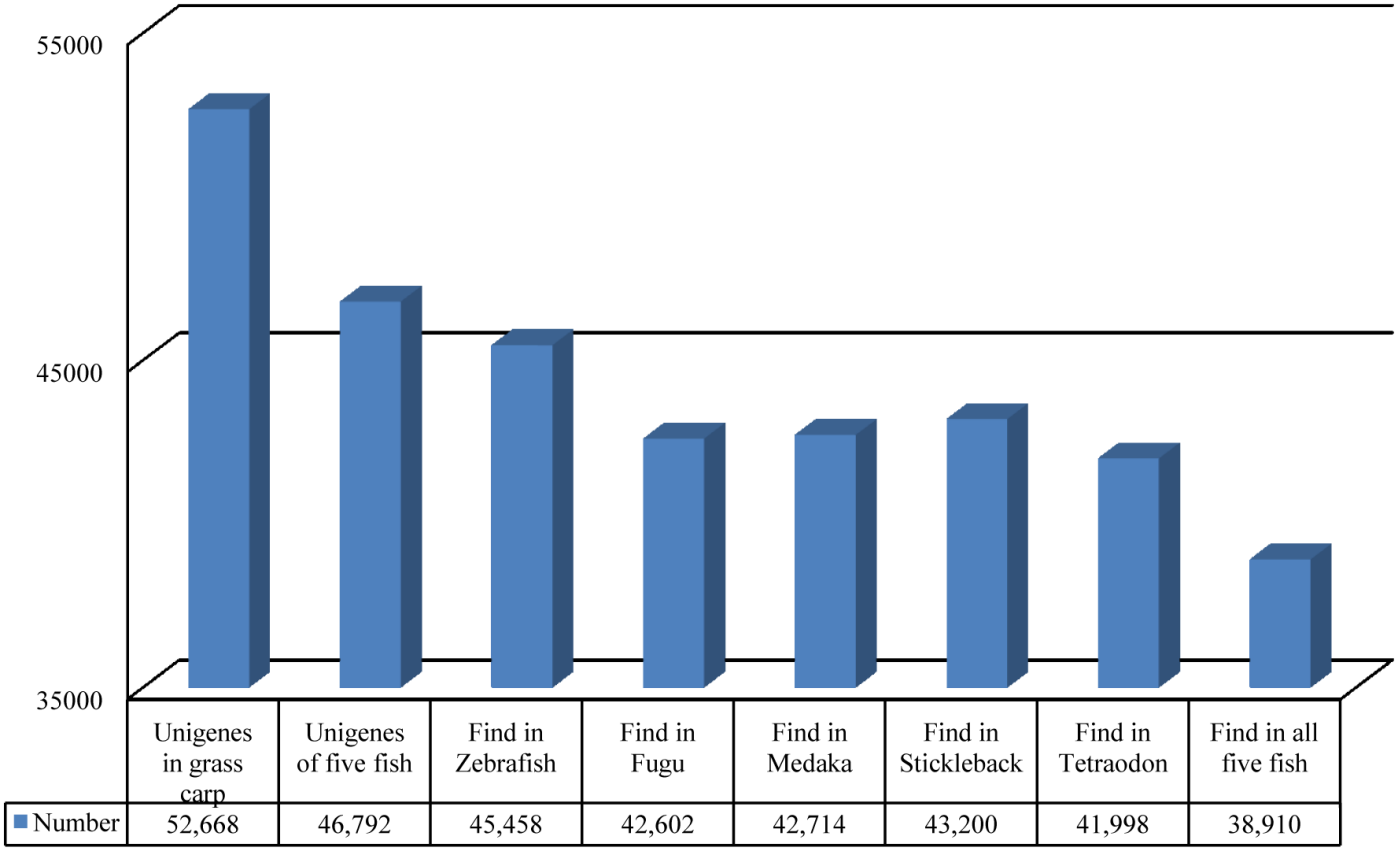
Genes conserved in *C*. *idella* and five model fish species (zebra fish, fugu, stickleback, medaka and *Tetraodon*). BlastX was used to identify *C*. *idella* homologous genes with other fish species by searching public database.

To evaluate the evolutionary conservation of the annotated unigenes, the sequences were compared with the databases of zebra fish (*Danio rerio*), fugu (*Takifugu rubripes*), stickleback (*Gasterosteus aculeatus*), medaka (*Oryzias latipes*) and a pufferfish (*Tetraodon sp*.), respectively (Table 2). A total of 38,910 (73.88%) putative known unigenes were found in all five species; 45,458 (86.31%) were found in the zebra fish database (Fig 2), indicating a high level of conservation of the genome resourses between *C*. *idella* and other teleost fish species, especially the zebra fish [51].

**Table 2:** Blast X search analysis of all transcripts of *C*. *idella*.

Refseq/Ensembl	Unigene hits*	Unique protein	Percentage of total unique proteins
Fugu	42,602*	21,246	44.41% of 47,841
Medaka	42,714*	16,569	67.15% of 24,674
Stickleback	43,200*	18,007	65.32% of 27,576
Tetraodon	41,998*	16,078	69.55% of 23,118
Zebrafish	45,458*	23,092	51.91% of 44,487

Note:

* Number of significant sequences (E-value < 1e-50) using all *C*. *idella* unigenes as queries to search against five databases.

### Unigenes annotation and classification

**Fig 3:**
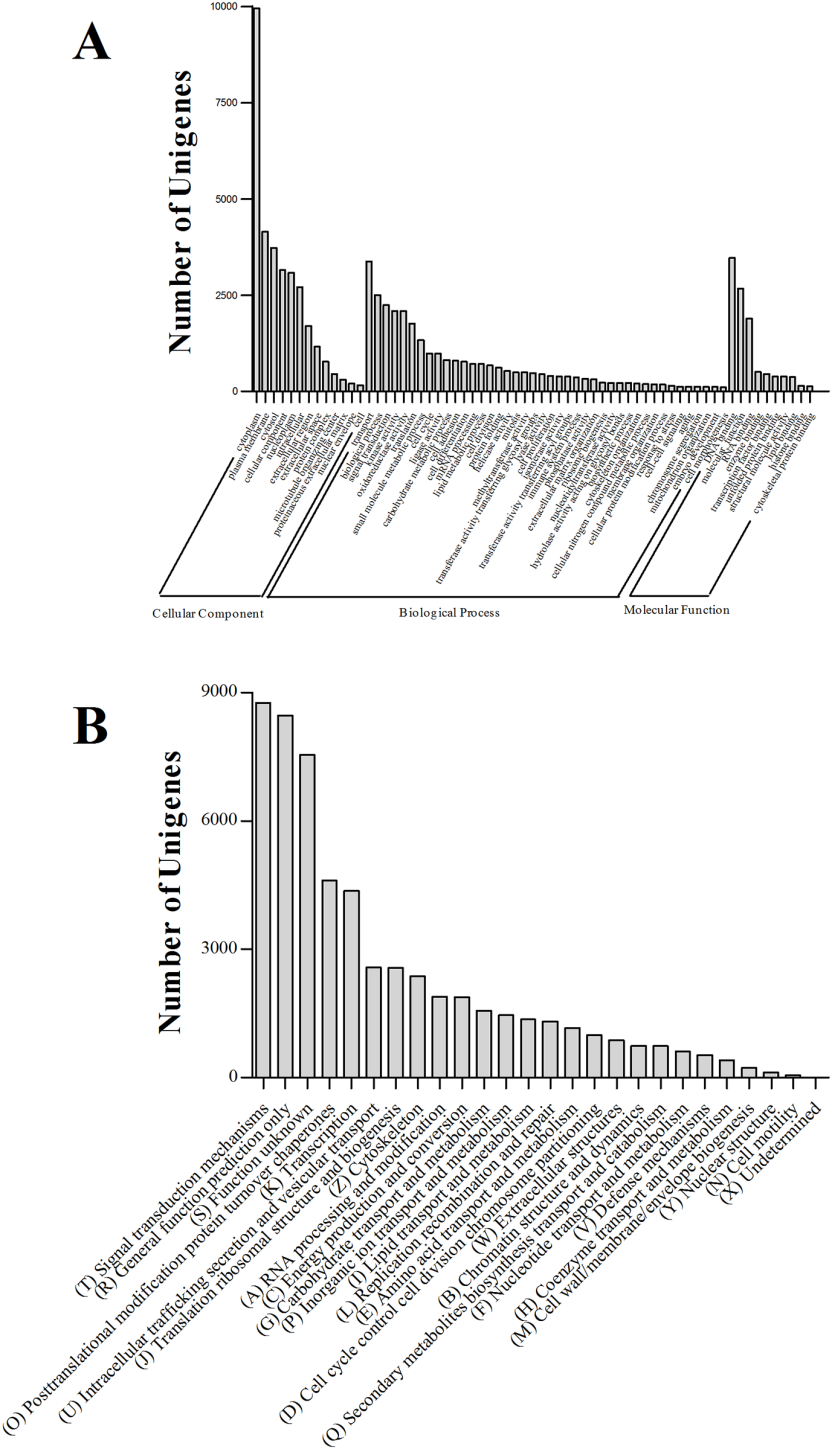
Functional annotation of *C*. *idella* Unigenes. (A) Go term distribution for the cellular component, biological process and molecular function categories. (B) eggNOG annotation.

A total of 52,668 unigenes (27.46% of all the transcripts) were assigned to known protein sequences (Table 3). 41,347 (78.50%) of the unigenes were annotated to three categories (“molecular function”, “cellular component” and “biological process”), with 90 functional groups, and 72,473 functional terms (Fig 3A). The “cellular components” category (containing 31,537 functional terms; 43.52%) was in the majority, followed by the “biological processes” category (30,347; 41.87%) and the number of functional terms in the “molecular functions” category (10,589; 14.61%) was the smallest. Among these functional groups, a large number of putative unigenes were involved in the “cytoplasm” (9,944), “plasma membrane” (4,144) and “cytosol” (3,726), indicating that the immune response in the grass carp involved cellular metabolic activities, which is consistent with the status of the fish. Other prominent functional groups also included “DNA binding” (3,467), “transport” (3,376), “cellular component” (3,158) and “nucleoplasm” (3,091) (Fig 3A).

**Table 3:** Statistics of the annotation results for the *C*. *idella* unigenes.

	All	Nr	KO	GO	KOG	Swiss-Prot	eggNOG
**Number of Genes**	191,795	52,668	10,134	41,347	49,975	46,392	22,785
**% of Genes**	100	27.46	5.28	21.56	26.06	24.19	11.88

Note: Nr: NCBI non-redundant protein sequences, KO: KEGG Ortholog database, GO: Gene Ontology, KOG: Clusters of Orthologous Groups of proteins, Swiss-Prot: A manually annotated and reviewed protein sequence database, and eggNOG: evolutionary genealogy of genes: Non-supervised Orthologous Groups.

A total of 49,975 unigenes (26.06%) were matched to 25 eukaryotic orthologous groups (annotated using the eggNOG Functional Category database; [52]) and all of them were determined (Fig 3B). The largest category was “signal transduction mechanisms” (8,765; 17.54%), consistent with other research [37, 53–55]; this showed that the unigenes were mostly involved in regulating signal transduction to achieve a new balanced environment *in vivo*, after *A*. *hydrophila* stimulation. “General function prediction only” was the second largest category (8,463; 14.78%). 13.19% of the unigenes were assigned to the “function unknown” category, indicating that the 7,551 unigenes, which included complement component genes, were involved in unknown mechanisms in *C*. *idella* (Fig 3B). Additionally, the relative enrichment categories included “post-translational modification, chaperone” (4,615; 8.06%), “transcription” (4,363; 7.62%), “intracellular trafficking, secretion, and vesicular transport” (2,585; 4.51%), “translation, ribosomal structure and biogenesis” (2,573; 4.49%) and “cytoskeleton” (2,373; 4.14%) (Fig 3B).

**Fig 4:**
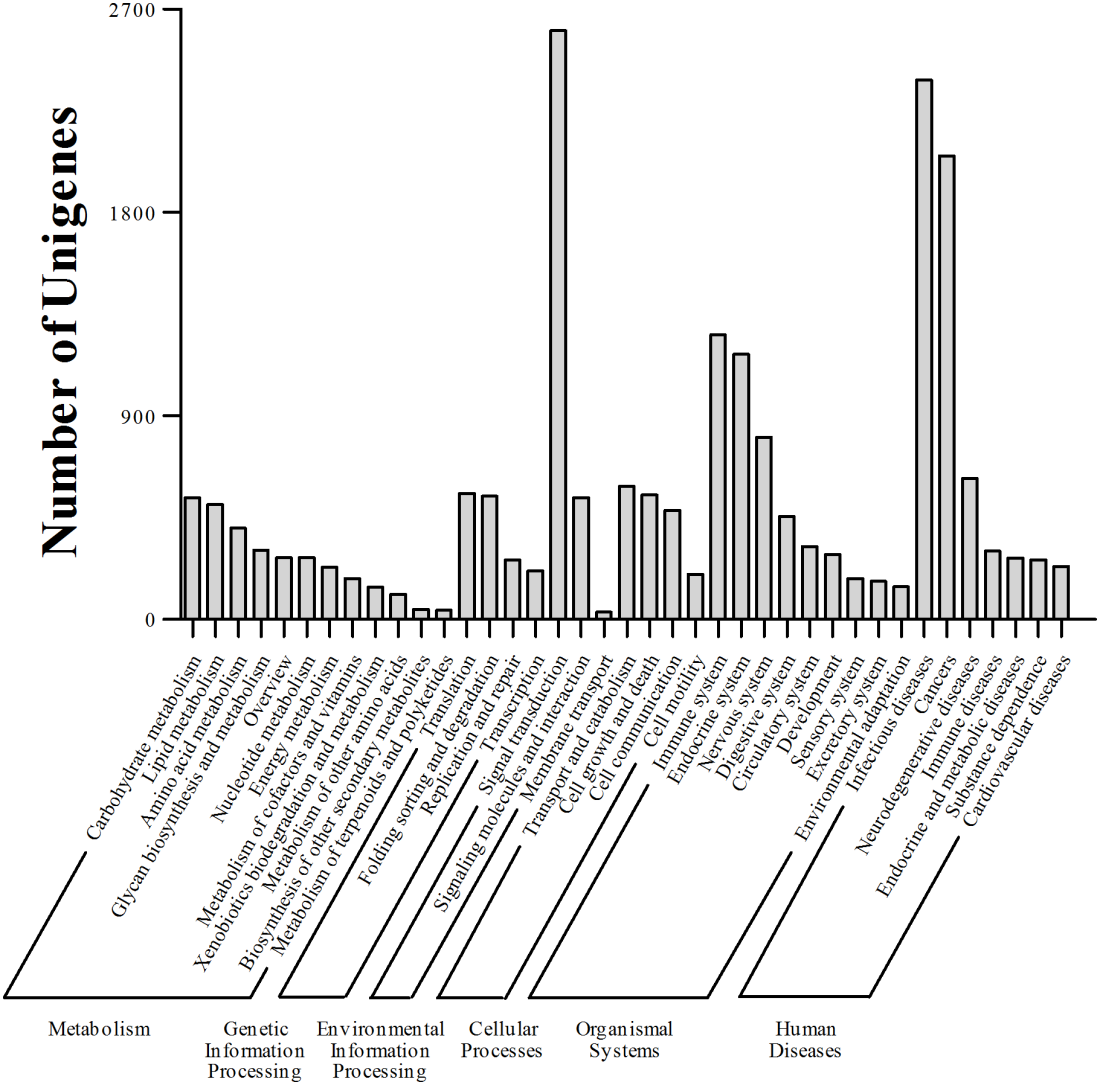
KEGG pathway in hierarchy 2. Unigenes were classified in metabolism, genetic information processing, environmental information processing, cellular processes, organismal systems and human diseases categories.

Through functional classification of the unigenes by exploring the pathways in the spleen of the grass carp [56, 57], 20,569 unigenes were assigned to six main categories; these included “human diseases” (6,136 of the assigned unigenes; 29.83%), “organismal systems” (4,798; 23.33%), “environmental information processing” (3,176; 15.44%), “metabolism” (3,054; 14.85%), “cellular processes” (1,824; 8.87%) and “genetic information processing” (1,581; 7.69%). Among the 38 pathways in hierarchy 2, the most represented were the “signal transduction” (2,605) and the “infectious diseases” (2,387) pathways (Fig 4). In particular, some immune-related pathways were also identified, such as the “complement and coagulation cascades” (65) and the “chemokine signaling” (149) pathways [58] (Table 4). These results indicate that an abundance of unigenes might have been involved in disease-resistance and the immune response, consistent with the status of the tested samples.

**Table 4:** Top 15 list of pathways related to immune system.

Pathway Hierarchy 2	KEGG Pathway	Unigene Number
Immune system	Chemokine signaling pathway	149
Immune system	Platelet activation	131
Immune system	Leukocyte transendothelial migration	119
Immune system	T cell receptor signaling pathway	111
Immune system	Fc gamma R-mediated phagocytosis	107
Immune system	Toll-like receptor signaling pathway	83
Immune system	Natural killer cell mediated cytotoxicity	78
Immune system	B cell receptor signaling pathway	75
Immune system	Complement and coagulation cascades	65
Immune system	Antigen processing and presentation	58
Immune system	RIG-I-like receptor signaling pathway	55
Immune system	Fc epsilon RI signaling pathway	55
Immune system	Hematopoietic cell lineage	50
Immune system	Cytosolic DNA-sensing pathway	47
Immune system	NOD-like receptor signaling pathway	46

### Expression and classification of the differentially expressed genes

**Fig 5:**
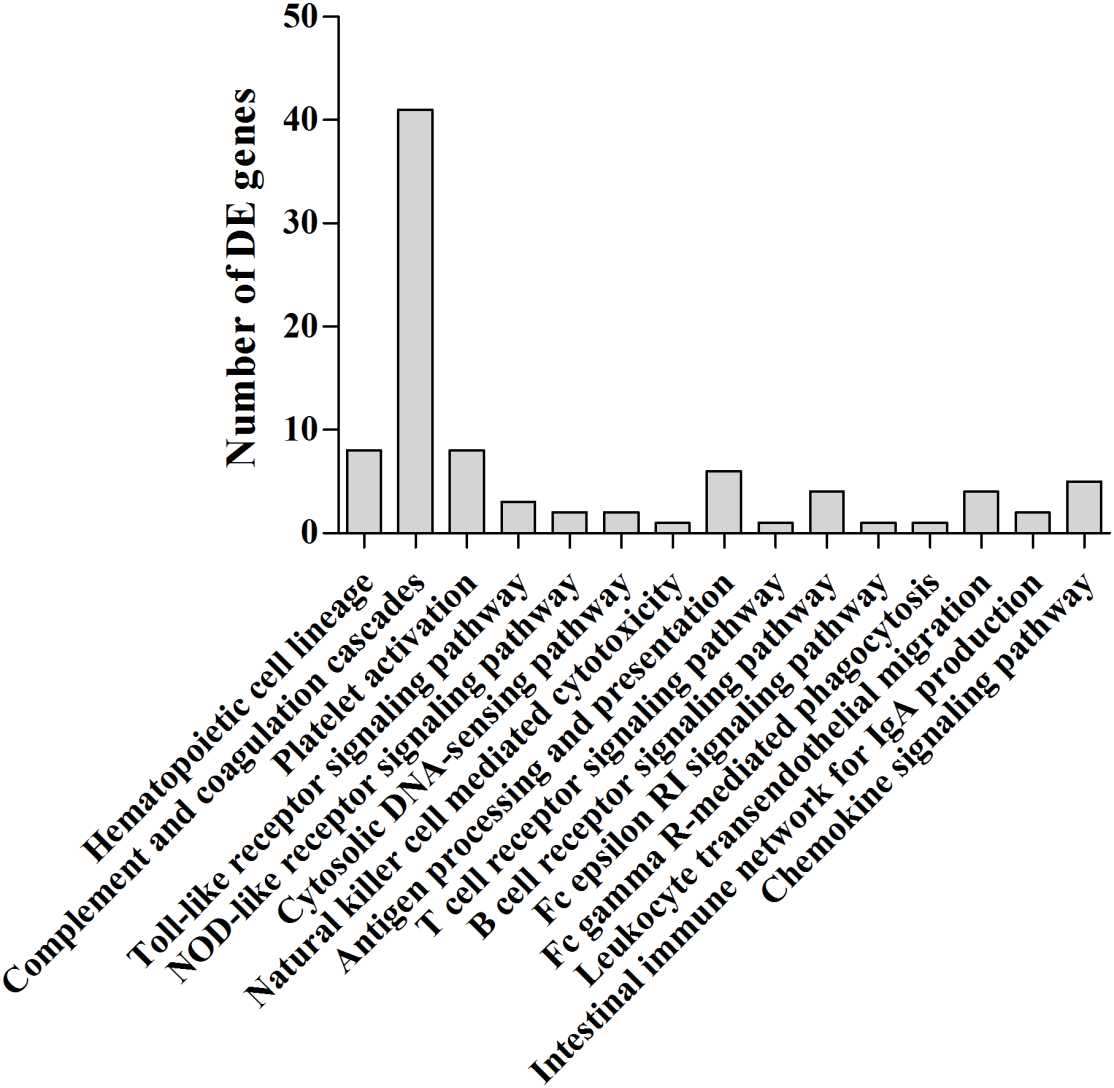
DE genes related to immune system.

A total of 2,992 DEGs (those that were at least 2-fold up- or down-regulated, with a *p*-value <0.05) were found by comparing each of the six experimental groups with the control group. Specific and shared DEGs were classified into 39 pathways, of which 89 putative DEGs were involved in the immune system (Table 5). Of those involved in the immune system, 41 (46.07%) were matched to the “complement and coagulation cascades” pathway, and 8 (8.99%) each were classified into the “hematopoietic cell lineage” and “platelet activation” pathways (Fig 5). The results indicate that complement components play an important role in fighting against bacterial infection.

**Table 5:** DE genes pathways classification of *C*. *idella*.

Category	Pathway	Genome	DE Gene	P-value	-Log10(P-Value)
Metabolism	Overview	274	36	1.05E-10	9.98
	Carbohydrate metabolism	538	70	3.27E-19	18.49
	Energy metabolism	231	16	1.54E-02	1.81
	Lipid metabolism	508	92	5.33E-36	35.27
	Nucleotide metabolism	274	21	1.91E-03	2.72
	Amino acid metabolism	403	63	1.53E-21	20.82
	Metabolism of other amino acids	111	21	1.11E-09	8.96
	Glycan biosynthesis and metabolism	307	12	0.50	0.30
	Metabolism of cofactors and vitamins	182	38	7.5E-18	17.12
	Metabolism of terpenoids and polyketides	41	5	0.019	1.73
	Biosynthesis of other secondary metabolites	43	10	3.61E-06	5.44
	Xenobiotics biodegradation and metabolism	142	54	2.03E-39	38.70
Genetic Information Processing	Transcription	215	5	0.91	0.040
	Translation	556	54	3.89E-10	9.41
	Folding, sorting and degradation	545	17	0.82	0.084
	Replication and repair	265	7	0.88	0.057
Environmental Information Processing	Membrane transport	33	4	0.035	1.46
	Signal transduction	2,605	112	8.94E-02	1.05
	Signaling molecules and interaction	538	31	0.014	1.84
Cellular Processes	Transport and catabolism	590	38	0.0011	2.93
	Cell motility	202	3	0.98	0.0072
	Cell growth and death	550	8	0.99	0.00015
	Cell communication	482	19	0.46	0.34
Organismal Systems	Immune system	1,262	89	0.28	0.55
	Endocrine system	1,174	77	2.79E-06	5.55
	Circulatory system	322	20	0.022	1.66
	Digestive system	455	63	8.66E-19	18.06
	Excretory system	169	13	0.012	1.91
	Nervous system	806	36	0.18	0.76
	Sensory system	180	11	0.082	1.09
	Development	286	14	0.20	0.70
	Environmental adaptation	143	6	0.46	0.34
Human Diseases	Cancers	2,051	99	0.0085	2.07
	Immune diseases	303	17	7.06E-02	1.15
	Neurodegenerative diseases	625	26	0.34	0.47
	Substance dependence	264	14	1.30E-01	0.88
	Cardiovascular diseases	235	4	0.98	0.0092
	Endocrine and metabolic diseases	271	11	0.45	0.35
	Infectious diseases	2,387	81	0.86	0.065

The hierarchical clusters separate the DEGs according to their expression levels (S2 Fig). Those genes in control group (0 h) had minimum expression values and were most closely clustered with those in the 4 h group, followed by the 12, 8, 24, 72 and 48 h groups, in that order; but, there was a large distance between the 0 and 4 h groups, and the other groups. The volcano plots show the difference in expression levels between the DEGs in the control group (0 h) and those in each other group; the blue dots represent the number of up- or down-regulated genes (with fold changes >2 and *p*-values <0.05; S3 Fig). Compared with the control group (0 h), there were 195 up-regulated DEGs at 4 h, 744 at 8 h, 408 at 12 h, 1,195 at 24 h, 1,026 at 48 h and 1,578 at 72 h.

### Gene expression in the complement and coagulation cascades pathway

**Fig 6:**
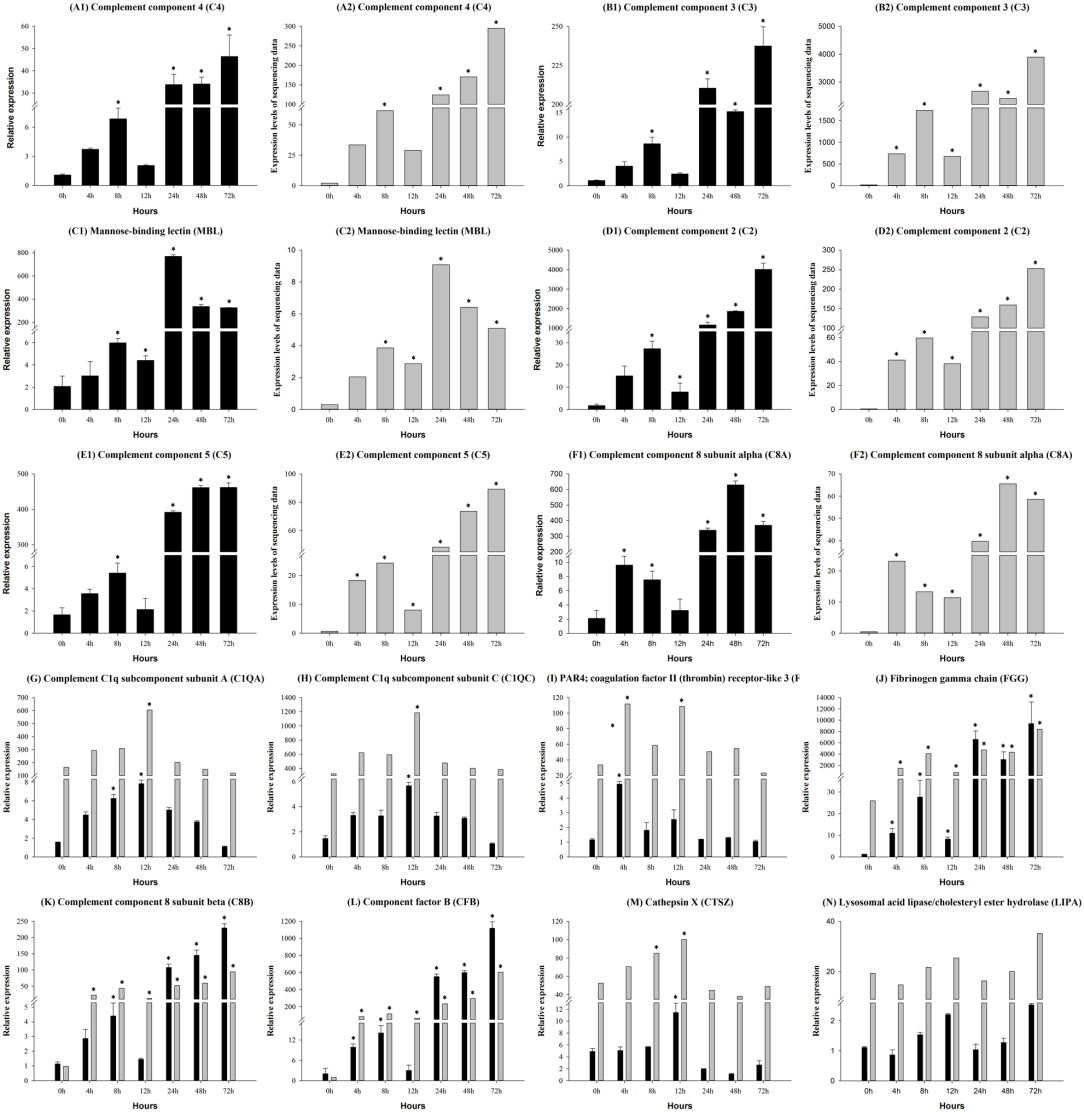
Complement and coagulation cascades pathway gene expression in control and experimental group intra-peritoneal injection with 2.7×10^7^ CFU/mL (100 μL/fish) *Aeromonas hydrophila*, as determined by qRT-PCR. (A1 and A2) C4, (B1 and B2) C3, (C1 and C2) MBL, (D1 and D2) C2, (E1 and E2) C5, (F1 and F2) C8A, (G) C1QA, (H) C1QC, (I) F2R, (J) FGG, (K) C8B, (L) CFB, (M) CTSZ, and (N) LIPA. The black and the gray column represent selected gene and sequencing data, respectively. Values are presented as the mean ± SD (n = 6). The expression levels with * indicate significant differences with control group (p<0.05).

In the present study, 16 candidate genes were selected from complement- and lysosome-pathway to validate the expression levels by qRT-PCR, and then compared with the RPKM values of the corresponding genes that were sequenced using the Illumina NextSeq^™^ 500 platform. The expression levels of the candidate genes followed the same trends as those observed in the RNA-Seq data (Fig 6). The complement C1q subcomponent subunit A (C1QA) gene was significantly up-regulated compared to the control between 8 h and 12 h (Fig 6G), and the complement C1q subcomponent subunit C (C1QC) gene was significantly up-regulated at 12 h (Fig 6H). Both the complement component 8 subunit beta (C8B) and the complement component factor B (CFB) (Fig 6K and 6L) genes were significantly up-regulated between 4 h and 72 h in the samples from the infected fish. The coagulation factor II (thrombin) receptor (F2R) gene was significantly up-regulated at 4 h and 12 h, compared to the control (Fig 6I). However, the fibrinogen gamma chain (FGG) gene was significantly down-regulated between 4 h and 72 h (Fig 6J). The regulation of the complement component 8 subunit alpha (C8A) gene was more irregular than the genes of the complement and coagulation cascades pathway. C8A was significantly up-regulated at 4 h and 8 h, but down-regulated at 12 h, and then, increased between 24 h and 72 h (Fig 6F1). Other genes with similar expression patterns includedthe complement component 2 (C2) (Fig 6D1 and 6D2), C3 (Fig 6B1 and 6B2), C4 (Fig 6A1 and 6A2), C5 (Fig 6E1 and 6E2) and mannose-binding lectin (MBL) (Fig 6C1 and 6C2) genes. In the antigen processing system, the cathepsin X (CTSX) gene was significantly up-regulated at 12 h, but down-regulated between 24 h and 72 h (Fig 6M), however, the lysosomal acid lipase/cholesteryl ester hydrolase (LIPA) gene was unaffected by the infection (Fig 6N).

The complement and coagulation cascades pathway has been studied at depth in mammals. Involved in the innate immune system, the complement components play important roles in anti-bacterial defenses [59–61]. Previous studies illustrated that teleost fish, like other vertebrates, contain three complement pathways, including a classical pathway, alternative pathway and lectin pathway [62]. The complement component C3, as a crucial factor in the complement pathways, is cleaved into C3a and C3b, and then to C5, to form the membrane attack complex (MAC). Some studies have indicated that the classical and alternative pathways in teleost fish, as the first line of defense, have a significant effect on pathogenic microorganism invasions [63, 64]. However, there is very little information about the lectin pathway in fish [65]. Complement component C4 plays an important role in activating downstream paths in the classical pathway [62]. In the present study, the expression level of C4 was affected in a time-dependent manner, by *A*. *hydrophila* infection. However, the expression results were opposite to those found in grouper (*Epinephelus coioides*) [66]. These differences may have been a result of the different fish species, or because the experiments were conducted at different developmental stages of the fish. In *C*. *idella*, most of the genes involved in the complement pathway were significantly up-regulated between 0 h and 4 h, illustrating that the pathway was activated during the early stages of the infection.

### Gene expression in the complement-related pathways

**Fig 7:**
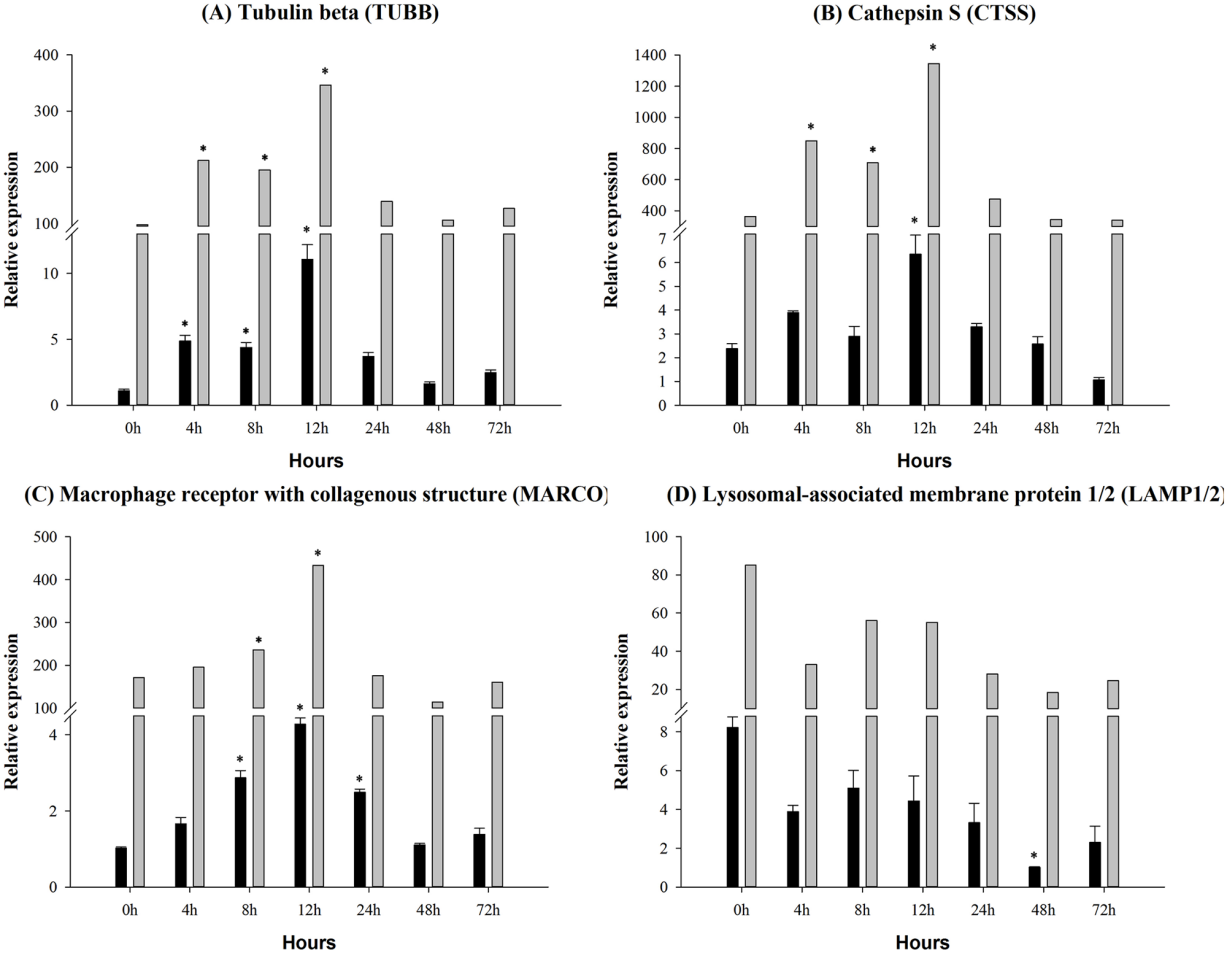
Phagosome-related pathway gene expression in control and experimental group intra-peritoneal injection with 2.7×10^7^ CFU/mL (100 μL/fish) *Aeromonas hydrophila*, as determined by qRT-PCR. (A) TUBB, (B) CTSS, (C) MARCO, (D) LAMP1/2. The black and the gray column represent selected gene and sequencing data, respectively. Values are presented as the mean ± SD (n = 6). The expression levels with * indicate significant differences with control group (p < 0.05).

**Fig 8:**
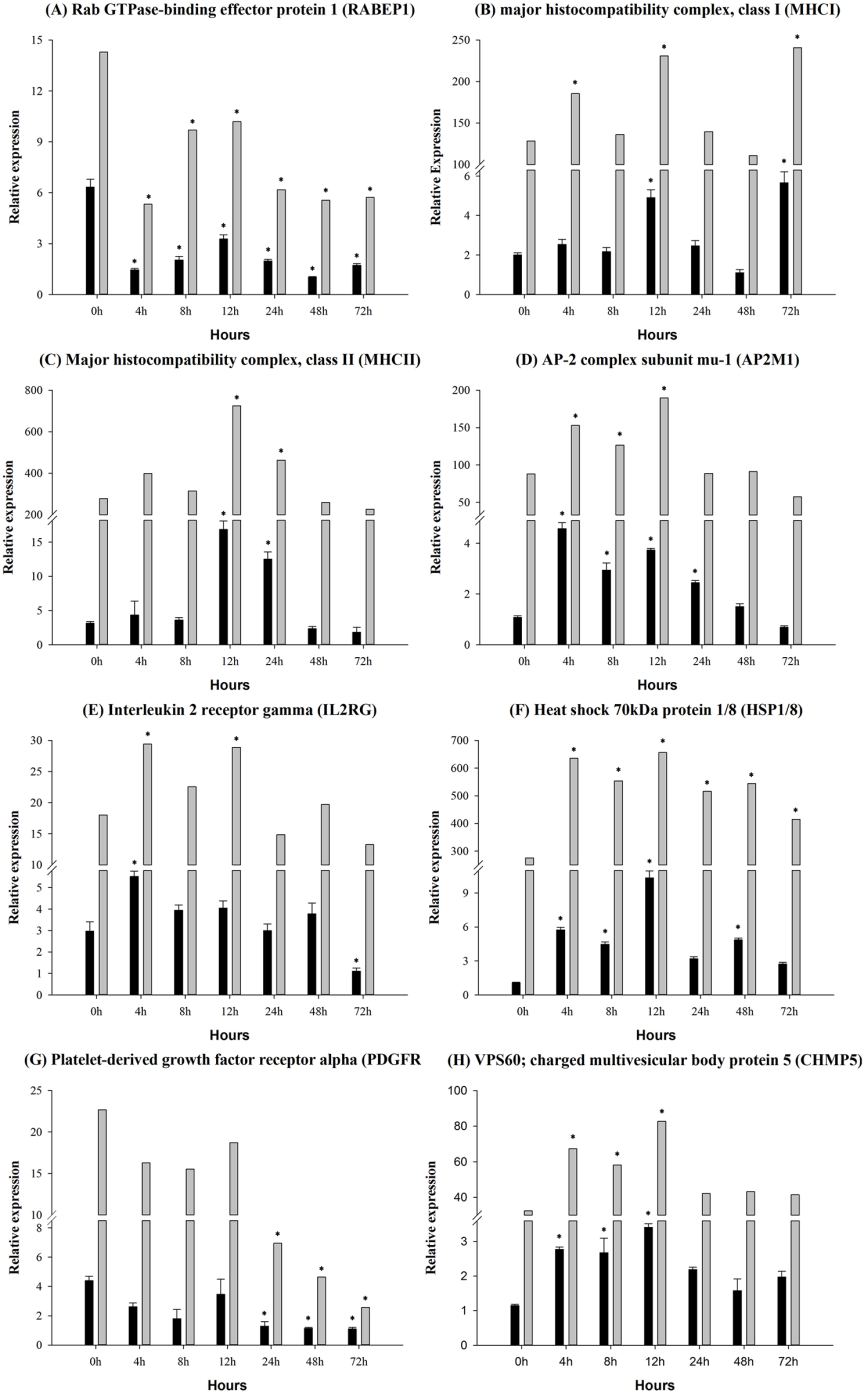
Endocytosis-related pathway gene expression in control and experimental group intra-peritoneal injection with 2.7×10^7^ CFU/mL (100 μL/fish) *Aeromonas hydrophila*, as determined by qRT-PCR. (A) RABEP1, (B) MHCI, (C) MHCII, (D) AP2M1, (E) IL2RG, (F) HSP1/8, (G) PDGFRA, (H) CHMP5. The black and the gray column represent selected gene and sequencing data, respectively. Values are presented as the mean ± SD (n = 6). The expression levels with * indicate significant differences with control group (p < 0.05).

**Fig 9:**
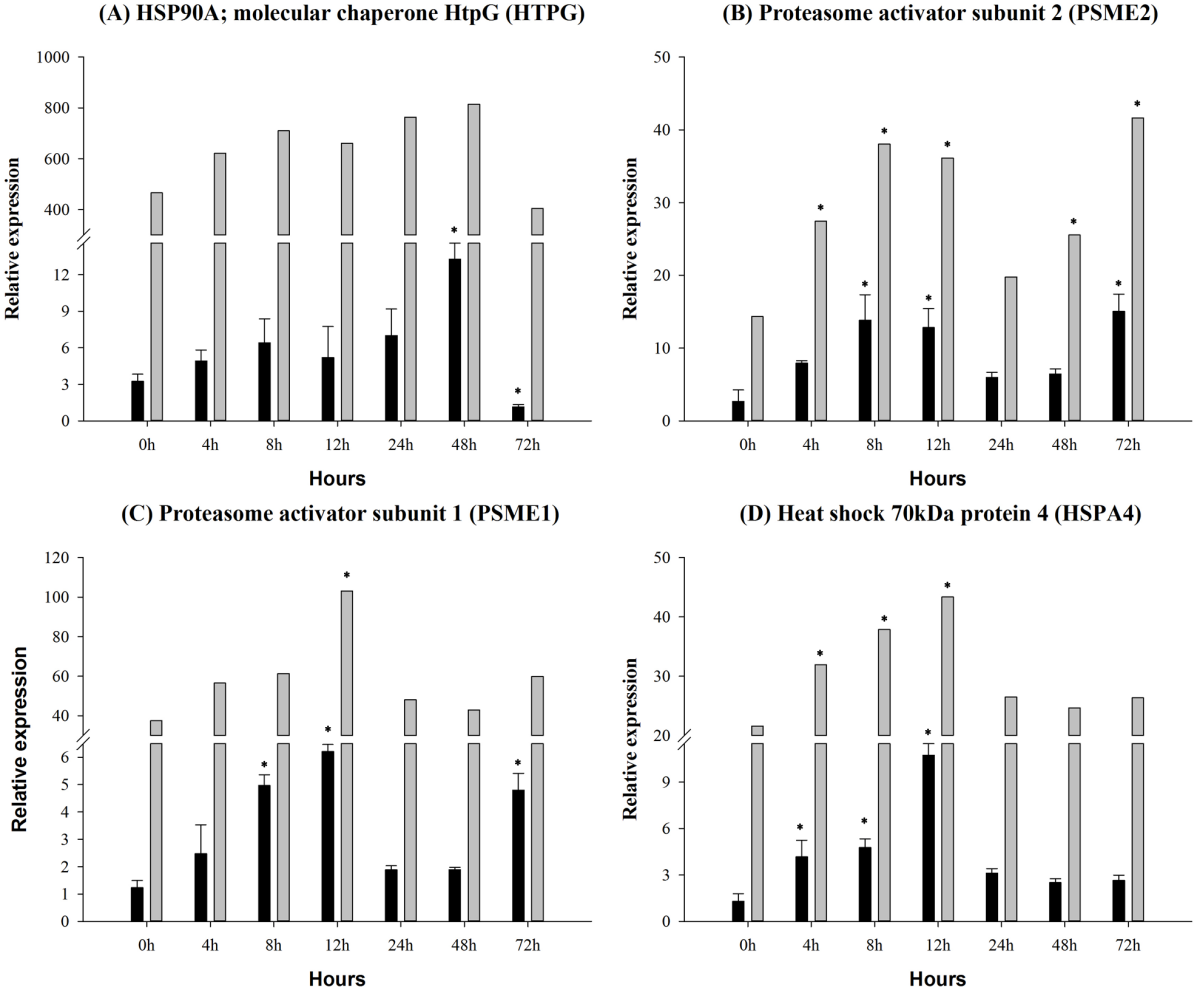
Antigen processing system gene expression in control and experimental group intra-peritoneal injection with 2.7×10^7^ CFU/mL (100 μL/fish) *Aeromonas hydrophila*, as determined by qRT-PCR. (A) HTPG, (B) PSME2, (C) PSME1, (D) HSPA4. The black and the gray column represent selected gene and sequencing data, respectively. Values are presented as the mean ± SD (n = 6). The expression levels with * indicate significant differences with control group (p<0.05).

In order to intuitively understand the effects of infection on the innate immune system, we considered the phagosome (Fig 7), endocytosis (Fig 8), antigen processing (Fig 9) pathway, because these are the complement-related pathways. The expression levels of 18 DEGs were measured by qRT-PCR, which showed corresponding results with the sequencing data. The tubulin beta (TUBB) gene was significantly up-regulated between 4 h and 12 h (Fig 7A). The macrophage receptor with collagenous structure (MARCO) gene was significantly up-regulated at 8 h and 12 h (Fig 7C). The protein cathepsin S (CTSS) gene was significant increased at 12 h (Fig 7B). The major histocompatibility complex, class I (MHCI) gene was significantly up-regulated at 4 h, 12 h and 72 h (Fig 8B). The charged multivesicular body protein 5 (CHMP5) gene was up-regulated between 4 h and 12 h by the infection (Fig 8H). In contrast, the lysosomal-associated membrane protein 1/2 (LAMP1/2) gene was unaffected in the infection samples (Fig 7D). Rab GTPase-binding effector protein 1 (RABEP1) gene (Fig 8A) and the platelet-derived growth factor receptor alpha (PDGFRA) gene were significantly down-regulated (Fig 8G). The interleukin 2 receptor gamma (IL2RG) gene expression level was increased at 4 h and 12 h, and significantly down-regulated at 72 h (Fig 8E). The major histocompatibility complex, class II (MHCII) gene was significantly elevated at 12 h and 24 h (Fig 8C), that of the heat shock 70kDa protein 1/8 (HSP1/8) gene between 4 h and 12 hand the AP-2 complex subunit mu-1 (AP2M1) gene between 4 h and 12 h (Fig 8F and 8D). The molecular chaperone (HTPG) gene was significantly up-regulated at 48 h and down-regulated at 72 h (Fig 9A). The expression level of proteasome activator subunit 2 (PSME2) gene and the proteasome activator subunit 1 (PSME1) gene were significantly elevated at 8 h and 12 h by the *A*. *hydrophila* infection (Fig 9B and 9C). The heat shock 70kDa protein 4 (HSPA4) gene occurred between 4 h and 12 h (Fig 9D).

As described above, the expression levels of the TUBB gene increased between 0 h and 4 h; we hypothesize that bacterial infection of *C*. *idella* initially resulted in the activation of the complement components, causing cells to up-regulate their expression of the tubulin gene. This would have allowed transport of the suddenly increased amount of complement pathway-related proteins out of the cells. The MARCO gene is involved in phagocytosis; some studies have indicated that lipopolysaccharides (LPS) can induce autophagy and bacteria have been found in macrophages [67]. In the present study, the MARCO gene expression was up-regulated at 12 h. However, most complement pathway-related genes were down-regulated at 12 h, such as C2, C3, C4, C5 and C8A. This was probably because of the activation of phagocytosis, which would have followed the identification of the bacteria by the complement system. The results illustrate that a putative regulatory network may exist in the innate immune system.

## Conclusions

In this study, we performed high-throughput sequencing of spleen tissue from *C*. *idella* specimens infected by *A*. *hydrophila*. 52,668 unigenes and 2,992 DEGs were obtained from the transcriptome data. 89 DEGs were annotated as part of the immune system. The results indicate that the response of grass carp to *A*. *hydrophila* infection depends on the complement and coagulation cascades pathways. Additionally, complement-related pathways are involved in the defense against bacterial infection. The present study also provides evidence of the immune mechanisms in teleost fish. Improvements in activation of the complement components in *C*. *idella* would likely benefit their anti-bacterial defenses. Our study not only provides useful data for further studies on the innate immune system, but also facilitates improving disease resistance breeding for *C*. *idella*.

## Supporting Information

S1 Fig Length distribution of the sequencing. (TIF)

S2 Fig Hotmap and dendrogram of DE genes. (TIF)

S3 Fig Volcano plot of pairwise comparison of seven transcriptomes. (TIF)

S1 Table All selected genes and primers used for quantitative real-time PCR. (PDF)

S2 Table Characterization of raw data. (PDF)

S3 Table Characterization of clean data. (PDF)
