# Supplementary figures and images for: Transcriptome Analysis of the Innate Immunity-Related Complement System in Spleen Tissue of Ctenopharyngodon idella Infected with Aeromonas hydrophila
Source: PLoS One. 2016 Jul 6;11(7):e0157413. doi: 10.1371/journal.pone.0157413 (PMC4934786; doi:10.1371/journal.pone.0157413)

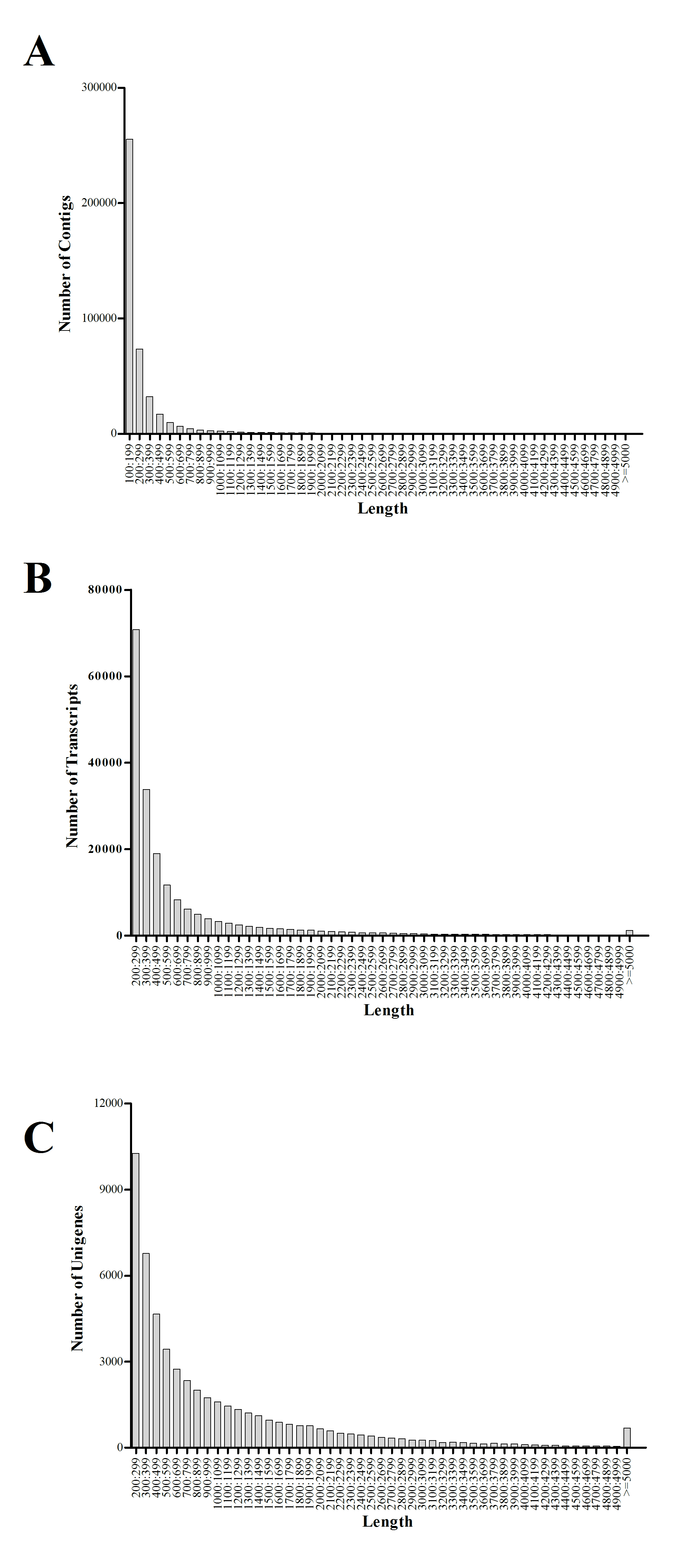

Supplement: S1 Fig — (TIF) [file pone.0157413.s001.tif]

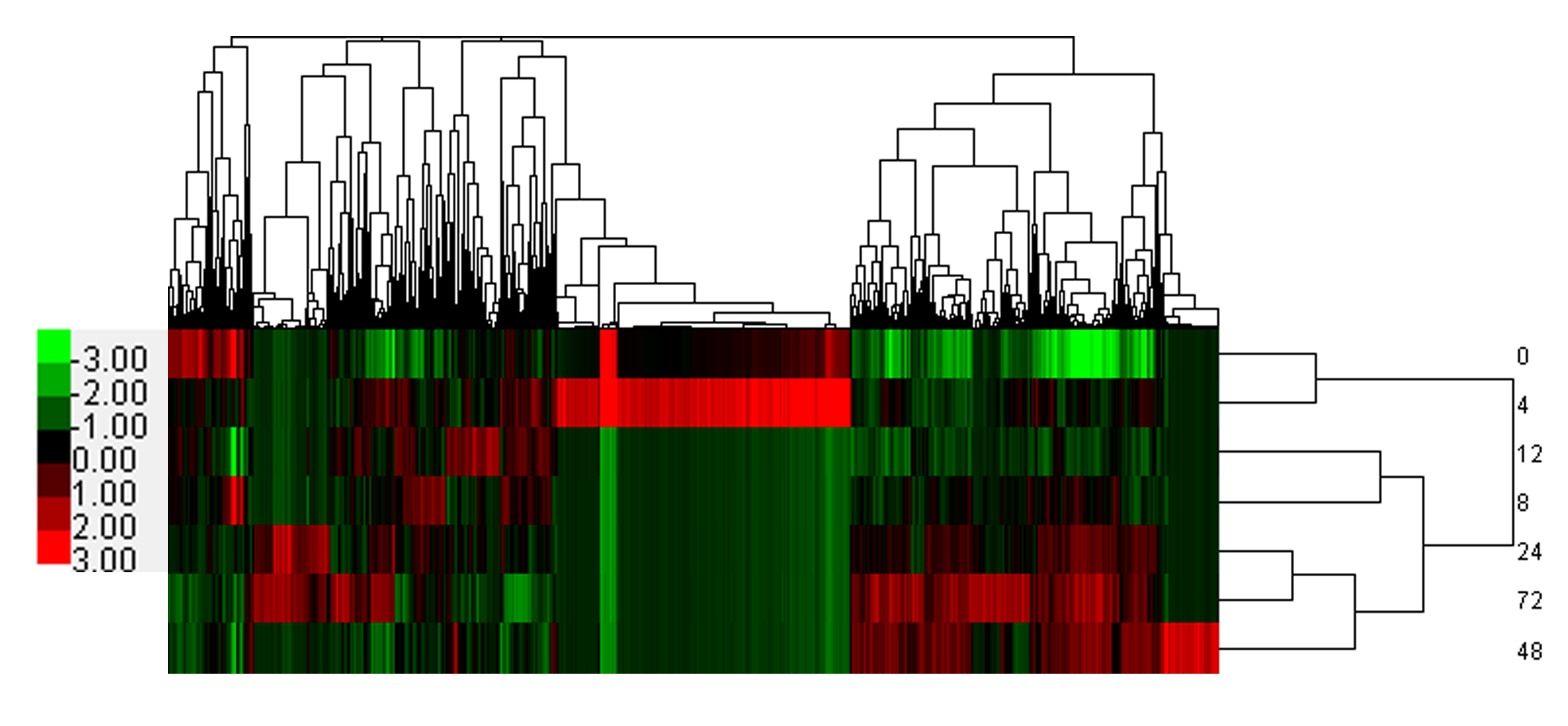

Supplement: S2 Fig — (TIF) [file pone.0157413.s002.tif]

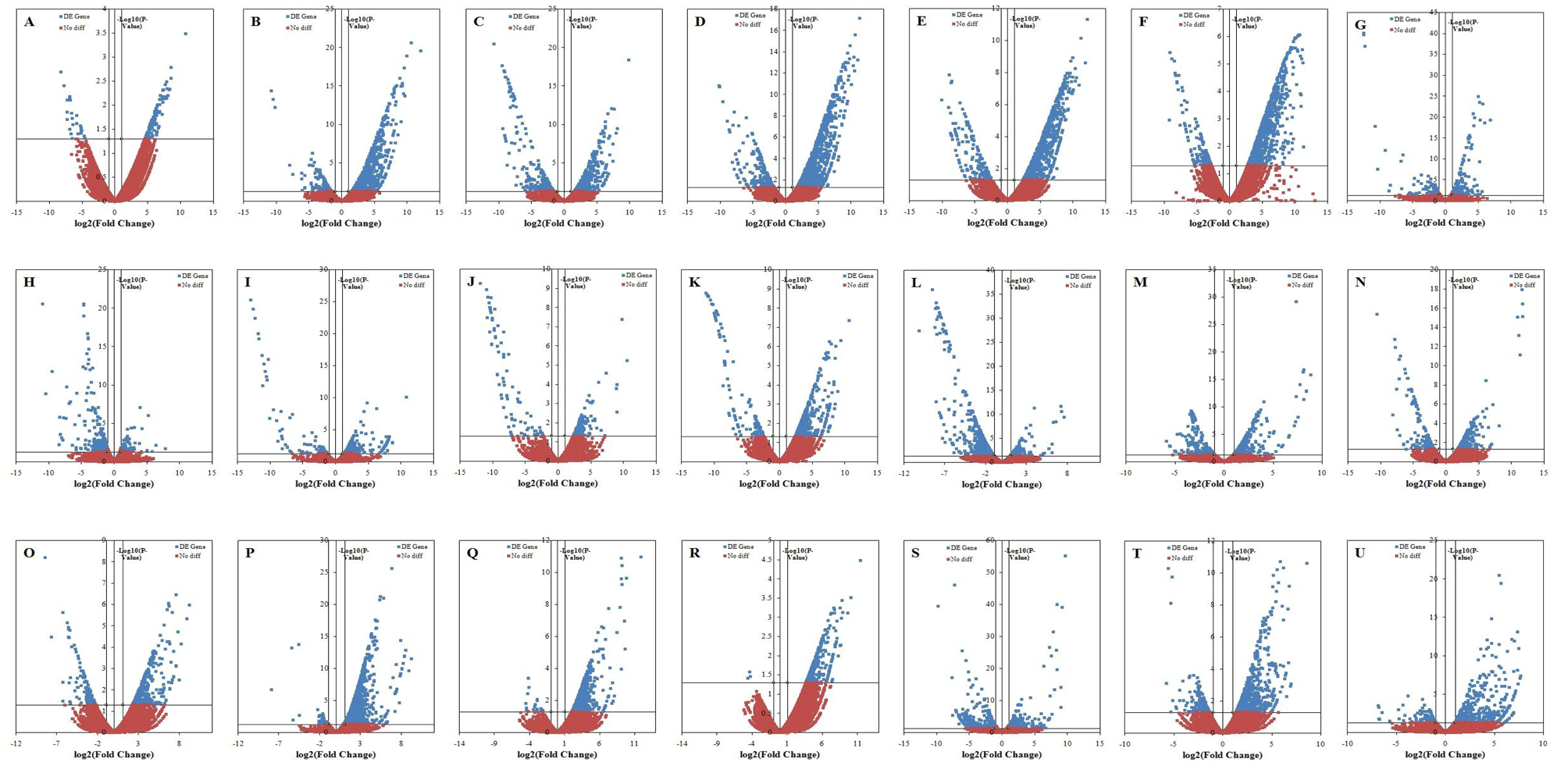

Supplement: S3 Fig — (TIF) [file pone.0157413.s003.tif]
