# Supplementary material for: Transcriptome Analysis of the Innate Immunity-Related Complement System in Spleen Tissue of Ctenopharyngodon idella Infected with Aeromonas hydrophila
Source: PLoS One. 2016 Jul 6;11(7):e0157413. doi: 10.1371/journal.pone.0157413 (PMC4934786; doi:10.1371/journal.pone.0157413)
Supplement: S1 Table — (PDF) [file pone.0157413.s004.pdf]

Gene name	Primer name	Primer sequence (5'- 3')
18S rRNA	18S rRNA-F, 18S rRNA-R	ATTTCCGACACGGAGAGG, CATGGGTTTAGGATACGCTC
β-actin	*β*-actin-F, *β*-actin-R	TCCTTCTTGGGTATGGAGTCTTG, CAGAGTATTTACGCTCAGGTGGG
HTPG	HTPG-F, HTPG-R	TGGACAGTGGCAAGGACC, GTTGTTTTAGTGATGACCGT
PSME1	PSME1-F, PSME1-R	ATGAAGGTCCTCCCTGTGGT, TCTGTGTCTGGAATCCCTCG
PSME2	PSME2-F, PSME2-R	GGATAGAAAACTACCGCCAG, CTCCATCTCCTCATCGTCAG
HSPA4	HSPA4-F, HSPA4-R	ATGCTGTAGAAATAGTGGGCG, CAGAGAGATGGGAAACGGAA
TUBB	TUBB-F, TUBB-R	CCTTCATCGGCAACAGCACT, ATTCGGACACCAGGTCGTTC
CTSS	CTSS-F, CTSS-R	GTGGTTCTTGTTGGGCGT, CTCTGGGTCTATTCCGCC
MARCO	MARCO-F, MARCO-R	TGCTGTGGTTCGTTTAGTGG, AAATGCTCCTCTCAGTTCCG
LAMP1/2	LAMP1/2-F, LAMP1/2-R	AGAAGACCTTGAGCGTTACACA, TGACTTCTGTTCCTGCCAATAA
CTSZ	CTSZ-F, CTSZ-R	AGTCAGTGGGCTGGACAAGA, TCCACTCTCATCAACACCCC
LIPA	LIPA-F, LIPA-R	ACATACTGAGCGTCAACCGAAT, GTTCCCACGACTGTTGCCTATC
RABEP1	RABEP1-F, RABEP1-R	AGTTGAAAGACGCCCTCG, AGTCCGCTTGCCGAATAC
MHCI	MHCI-F, MHCI-R	ATGCTACAGGTTTTTACCCCAG, TTCTCTGTCACACTCTTGCCCT
MHCII	MHCII-F, MHCII-R	TATCGCCCAAATGATGACGG, CAGCAGCCCCAGAGACAGAC
AP2M1	AP2M1-F, AP2M1-R	ATCGGTTGGCGTCGTGAAG, TGCTTCCCACTTTTCCCAG
IL2RG	IL2RG-F, IL2RG-R	CTCCTTCACTTTGCCTTTTCCCTC,AGGCAGGCGAGTATGAGGAGTATT
HSP1/8	HSP1/8-F, HSP1/8-R	CAGGACCACTCCAAGTTATG, CTTGGTCTCACCCTTGTATT
PDGFRA	PDGFRA-F, PDGFRA-R	ATAACTATCCCCTGCCTCGC, ACGCTGCTCTGAACTGCTTT
CHMP5	CHMP5-F, CHMP5-R	GTCCGTTGACAAGAAGATAGC, CTGGATGGTGTAGTTTGCTTG
C1QA	C1QA-F, C1QA-R	TTCTCTGTGTTGCGAAAAAG, TCCACCTGACACAACAAGAG
C1QC	C1QC-F, C1QC-R	GCATTCAGTGTGTCCCGT, CACCTGCTGGCTGCTCCT
F2R	F2R-F, F2R-R	CAAGCAAAACGATGAGTCCTAC, TCTGACACTTGAGAAAGGCGAG
MBL	MBL-F, MBL-R	CAACTTCAATCAGATGTCAAACACC, GCTCATATCTACAAAATGCCCTTCT
C3	C3-F, C3-R	CCGTCCTTCCGCTTCGTG, CCTCCTGCGGTGTGCGAC
C4	C4-F, C4-R	GGTGGTGGAGAGAACGCAG, CTTTTCGCTCGGTCCTCGC
FGG	FGG-F, FGG-R	TGTGGAGTTGCTGATTACCTAC, GTTAGCCTGTAGGATTGACTGC
C8A	C8A-F, C8A-R	ATACAACAATAAGGAGGTGGGCT, CAAGGCACTTTCCCATCTGTT
C8B	C8B-F, C8B-R	GTGGGGTGATGCTGTGTTCT, CCATTATTGAGACAGGGGGA
CFB	CFB-F, CFB-R	CCGAAAAACCCCAGAGTGC, GCCGAGCCACGGAACTTAT
C2	C2-F, C2-R	TCAGGGAGACCAAGAGAAATCAAT, TGCTTGTGAGATCTTTCCTGTTAA
C5	C5-F, C5-R	GTGCTGGCCTTATGTTCATTACAA, AGGTTTCAAGCGTCGGGATAC
