# Supplementary material for: Transcriptome Analysis of the Innate Immunity-Related Complement System in Spleen Tissue of Ctenopharyngodon idella Infected with Aeromonas hydrophila
Source: PLoS One. 2016 Jul 6;11(7):e0157413. doi: 10.1371/journal.pone.0157413 (PMC4934786; doi:10.1371/journal.pone.0157413)
Supplement: S2 Table — (PDF) [file pone.0157413.s005.pdf]

Sample	Reads	Raw reads	Raw Data (bp)	Q20 (%)	Q30 (%)	GC (%)	Reads Len. (bp)
0	R1	15,469,992	4,494,532178	90.22	80.47	53.04	150
	R2	15,469,992	4,494,532178				
	Paired	15,469,992	4,494,532178				
4	R1	19,595,381	5,639,822,783	90.64	81.21	51.98	150
	R2	19,595,381	5,639,822,783				
	Paired	19,595,381	5,639,822,783				
8	R1	31,707,453	9,212,432,680	88.66	78.13	52.08	150
	R2	31,707,453	9,212,432,680				
	Paired	31,707,453	9,212,432,680				
12	R1	23,391,956	6,875,501,359	87.75	77.01	51.79	150
	R2	23,391,956	6,875,501,359				
	Paired	23,391,956	6,875,501,359				
24	R1	23,228,368	6,772,712,000	90.44	80.92	52.28	150
	R2	23,228,368	6,772,712,000				
	Paired	23,228,368	6,772,712,000				
48	R1	19,792,133	4,857,778,347	85.80	76.28	61.83	150
	R2	19,792,133	4,857,778,347				
	Paired	19,792,133	4,857,778,347				
72	R1	16,329,387	4,816,250,934	90.01	80.09	50.84	150
	R2	16,329,387	4,816,250,934				
	Paired	16,329,387	4,816,250,934				
Average	Paired	21,359,238.57	6,095,575,754				
Total	Paired	149,514,670	42,669,030,281				
