# Supplementary material for: Transcriptome Analysis of the Innate Immunity-Related Complement System in Spleen Tissue of Ctenopharyngodon idella Infected with Aeromonas hydrophila
Source: PLoS One. 2016 Jul 6;11(7):e0157413. doi: 10.1371/journal.pone.0157413 (PMC4934786; doi:10.1371/journal.pone.0157413)
Supplement: S3 Table — (PDF) [file pone.0157413.s006.pdf]

Sample	Reads	Clean Reads	Clean Data (bp)	Useful Reads (%)	Useful Data (%)
0	R1	13,187,471	3,625,028,377	85.25	80.65
	R2	13,187,471	3,625,028,377		
	Paired	13,187,471	3,625,028,377		
4	R1	16,497,315	4,522,164,309	84.19	80.18
	R2	16,497,315	4,522,164,309		
	Paired	16,497,315	4,522,164,309		
8	R1	25,434,348	6,890,692,628	80.22	74.80
	R2	25,434,348	6,890,692,628		
	Paired	25,434,348	6,890,692,628		
12	R1	18,271,945	5,000,199,754	78.11	72.72
	R2	18,271,945	5,000,199,754		
	Paired	18,271,945	5,000,199,754		
24	R1	19,484,980	5,423,853,946	83.88	80.08
	R2	19,484,980	5,423,853,946		
	Paired	19,484,980	5,423,853,946		
48	R1	15,621,029	3,563,711,100	78.93	73.36
	R2	15,621,029	3,563,711,100		
	Paired	15,621,029	3,563,711,100		
72	R1	13,684,258	3,819,823,592	83.80	79.31
	R2	13,684,258	3,819,823,592		
	Paired	13,684,258	3,819,823,592		
Average	Paired	17,454,478	4,692,210,529		
Total	Paired	122,181,346	32,845,473,706		
